# Seasonal Variations of Faecal Cortisol Metabolites in Koalas in South East Queensland

**DOI:** 10.3390/ani11061622

**Published:** 2021-05-31

**Authors:** Flavia Santamaria, Rupert Palme, Rolf Schlagloth, Edith Klobetz-Rassam, Joerg Henning

**Affiliations:** 1 Koala Research-Central Queensland and Flora, Fauna and Freshwater Research, School of Health, Medical and Applied Sciences, Central Queensland University, North Rockhampton, QLD 4702, Australia; r.schlagloth@cqu.edu.au; 2 Department of Biomedical Sciences, University of Veterinary Medicine, 1210 Vienna, Austria; Rupert.Palme@vetmeduni.ac.at (R.P.); Edith.Klobetz-Rassam@vetmeduni.ac.at (E.K.-R.); 3 School of Veterinary Science, The University of Queensland, Gatton, QLD 4343, Australia; j.henning@uq.edu.au

**Keywords:** *Phascolarctos cinereus*, faecal cortisol metabolites, koala, baseline values, enzyme immunoassay, EIA

## Abstract

**Simple Summary:**

Koala habitat is threatened by urbanisation, agricultural activities and by increased temperatures and droughts caused by climate change. Habitat loss may cause stress in koalas, which, in turn, may exacerbate disease occurrence. Stress is associated with an increase of glucocorticoids, of which cortisol is the main one in most mammals. Cortisol is heavily metabolized and excreted via the faeces, where its metabolites can be measured, without interfering with the animal. However, before a link between events causing stress in koalas can be established, baseline levels in stress-free koalas need to be established. Our study has established which diagnostic procedure is best suited to measure these metabolites and has evaluated the physiological baselines levels in male and female koalas and in koalas during the breeding and non-breeding season.

**Abstract:**

The Koala (*Phascolarctos cinereus*) is an endemic marsupial inhabiting four states of Australia. Urbanisation, declining habitat, drought and fires are threatening the survival of this flagship species. These threats may cause acute and chronic stress in koalas, which might also be associated with occurrence of infectious diseases in koala populations. Stress may induce an increase in cortisol reflected in increased faecal cortisol metabolite (FCM) values. To be able to use faecal cortisol metabolites to measure stress levels in this species, our aim was to determine baseline values for males and females during breeding and non-breeding season. A total of 351 defecations were collected fortnightly, twice a day, for 12 months from koalas at a wildlife facility in South East Queensland. Samples were analysed with three different enzyme immunoassays (EIAs): a cortisol, 5α-pregnane-3β,11β,21-triol-20-one (37e) and tetrahydrocorticosterone (50c) EIA. The latter, which also reacts with tetrahydrocortisol, the main metabolite in koala faeces, was found to have the highest biological sensitivity and, therefore, is the most suitable EIA to measure stress levels in koalas. Utilising this EIA, we found significant differences (*p* < 0.05) in FCM values between males and females, breeding and non-breeding season, and between morning and evening samples. Values of faecal cortisol metabolites established in stress-free koalas in this study can serve as a reference for future studies in koalas.

## 1. Introduction

The Koala (*Phascolarctos cinereus)* is an endemic marsupial species, whose range spans throughout eastern and south eastern Australia. As many populations are declining, koalas have been listed as vulnerable in Queensland, the Australian Capital Territory and New South Wales under federal and state legislations, but are not listed in Victoria and South Australia [1].

Due to increased urbanisation along the east coast of Australia, anthropogenic activities, causing fragmentation and loss of habitat, are major threats [2,3,4] to this flagship species [5,6]. Moreover, koala populations are increasingly impacted by extensive clearing of habitat due to agriculture activities and mining, as well as droughts exacerbated by climate change [7,8]. Loss of habitat is an important stressor in wildlife often associated with the spread of infectious diseases [9], and this has also been documented in koalas [3]. Management initiatives, such as translocation, are implemented to mitigate the effect of habitat loss [10,11,12]. However, it has been reported that these activities may also potentially be responsible for both acute and chronic stress, increasing the likelihood of disease occurrence such as *Chlamydia* infection in koalas [13,14,15,16,17,18]. Thus, it is necessary to establish a suitable and reliable method to detect and measure stress levels and ultimately determine the impact of stress on the health of koalas [19,20].

Glucocorticoids (GCs), such as cortisol and corticosterone, are produced by the adrenal cortex and regulate several metabolic and physiological processes [21,22]. During acute or chronic stress events, GCs are part of a cascade of hormones involved in increasing the adrenocorticotropic hormone (ACTH) which, in turn, stimulates their increased release from the adrenal gland into the circulatory system [23,24,25].

Measuring GC values in plasma and saliva or GC metabolites in fur, urine and in faeces are various options to evaluate the stress response in wildlife and domestic animals [26,27,28]. However, blood sampling requires restraining animals and is, *per se*, a stressful event [23], and sampling saliva, urine and fur are not always practical options when dealing with many wildlife species. In contrast, faeces sampling for the evaluation of faecal GC metabolites, which includes faecal cortisol metabolites (FCMs), has become a popular non-invasive option used for many wildlife species [26,28].

Furthermore, in stress-free animals, physiological GCs concentration in plasma shows variations due to a circadian rhythm, characterised by peaks in the morning for diurnal species and peaks in the evening for nocturnal species, as well as episodic fluctuations may also exist during the day [22,24,26,29,30]. Therefore, using a non-invasive method, such as measuring the concentration of GC metabolites in faeces, ensures that physiological baseline values are reliably detected in stress-free animals. In many wild and domesticated mammalian species, including marsupials, FCM values have been used as a non-invasive method to measure stress [31,32,33].

To determine whether increases in plasma GCs are well reflected in FCMs, ACTH challenges were performed for many species [24,26]. These studies have demonstrated that the increase of GC levels in plasma is well reflected by an increase in FCMs [28,34]. Measuring the concentrations of FCMs represent the cumulative secretion of hormones, thus eliminating the issues of fluctuating values of plasma GCs obtained from blood samples [24,35].

The ACTH challenge has also been used in many studies on mammals [36,37] and marsupials [38,39], including koalas [40,41]. Earlier studies indicated a limited increase in plasma cortisol post challenge in one koala [42] and in a small number of koalas after potential stressful events [43] and, more recent research showed an increase in plasma cortisol levels, but not a corresponding increase of FCMs [44]. However, current studies have revealed an increase in plasma GCs and a correlation with FCM values after ACTH injection [38,39,40,41,45], albeit various levels of success and individual differences between animals.

Most cited studies on marsupials have used cortisol enzyme immunoassays (EIAs) to detect FCMs. However, due to differences in the metabolism and lag-time of excreted CMs among species [26], not one immunoassay can be used successfully to measure CMs in all marsupials, hence it is a prerequisite to thoroughly validate assays used to measure FCMs for each species [26,40,46]. During our previous study [47], we have characterised FCMs found in koalas’ faeces and have found a tetrahydrocorticosterone EIA (5β-pregnane-3α,11β,21-triol-20-one EIA; laboratory code: 50c EIA, first described by Quillfeldt and Möstl [48] and a 3β-allotetrahydrocorticosterone EIA (5α-pregnane-3β,11β,21-triol-20-one EIA; laboratory code 37e EIA; first described by Touma et al. [49]) to be well suited for measuring FCMs in this species.

To determine if FCM levels represent a response to a stressor, physiological baseline levels need to be established [24]. It was, therefore, our aim to determine baseline FCM values for male and female koalas, baseline FCM values during the breeding and non-breeding season of koalas and to explore if a diurnal rhythm in baseline FCM values exists. Based on our previous research [47], we evaluate FCM values using cortisol, 3β-allotetrahydrocorticosterone and tetrahydrocorticosterone EIAs, with the aim to identify which EIA has the highest biological sensitivity and therefore is most suitable to measure physiological baseline levels in koalas.

## 2. Materials and Methods

### 2.1. Koalas

This study was conducted at the wildlife park Wildlife HQ located at the Sunshine Coast, South East Queensland, Australia. A total of 8 female and 5 male captive koalas housed in outdoor enclosures were included in this study. Of these, 5 females and 4 males were sampled throughout the year-long project, while 1 male and 3 females were not available for the duration of the whole project and were replaced by other koalas of the same sex and age category. Therefore, defecations of 9 koalas were collected each fortnight. The mean (min–max) age of the koalas at the start of the study was 4.5 years (1.5–13 years). Wildlife HQ koalas are provided daily with freshly cut branches of eucalypt fodder of the same species and quality obtained locally from areas where wild koalas also live and water was provided *ad libitum.* The health of the koalas was monitored according to procedures of Wildlife HQ.

### 2.2. Faecal Sample Collection Regime

We placed rubber hollowed mats at the base of the tree stumps, where koalas were sitting, to separate the pellets from soil and to avoid contamination with urine. Fresh faecal pellets were collected from the animals immediately after each defecation. A total of 351 fresh defecations were collected fortnightly over a 12-month period (September 2019–August 2020). To ensure that pellets were collected fresh from each animal, researchers observed each koala until defecations were completed. Defecations were collected between 7:00 and 9:30 and between 15:00 and 18:00. Our previous study [47] showed that, after a cortisol injection, FCMs were excreted with a delay of around 10 h in both females and males. Therefore, FCM values obtained from pellets collected between 7:00 and 9:30 reflected *evening* plasma cortisol values, and FCM values obtained from pellets collected between 15:00 and 18:00 represented *morning* plasma cortisol values.

Due to disruptions caused by COVID-19, the number of fortnight samplings were reduced from 26 to 23 and one morning collection was missed for all koalas. A few collections were also missed for individual koalas. Fresh pellets were placed into sample tubes and stored at −20 °C in a portable freezer for transport and then stored at −80 °C until sample preparation and further analysis.

### 2.3. Sample Preparation, Extraction and EIAs

We followed the extraction procedure described in Palme et al. [50]. We used pestle and mortar to crush 2 frozen (wet) pellets, then placed 500 mg into a 10 mL tube and added 5 mL of 80% methanol. Samples were shaken for 30 min with an orbital rotator shaker, vortexed for 2 min on a hand vortex and centrifuged at 2500 RPM for 15 min. Completely dried down aliquots (0.25 mL) of the extracts in 1 mL Eppendorf tubes, sealed with paraffin film, were shipped to the University of Veterinary Medicine (Austria) for analysis. After redissolving the extracts in 80% methanol and a further dilution step with assay buffer (1 + 9), aliquots were analysed in duplicate with three EIAs. Table 1 shows information on these EIAs and the specific structures measured by each.

Details of the assays, including cross-reactions, have been published previously [47,49,50]. The 3 EIAs were selected based on the findings of Santamaria et al. [47] and used here to validate their biological sensitivity in evaluating FCM changes between breeding and non-breeding seasons, as well as between morning and afternoon. Intra-assay and inter-coefficients of variation (CVs) were below 10% and 15%, respectively, for a high and low concentration pool sample in all three assays. FCM levels are expressed as ln-transformed ng/g wet faeces.

### 2.4. FCM Variation within Defecations

In addition, between 7 and 13 single pellets from separate defecations of 10 koalas were analysed using all three EIAs to establish whether concentrations of FCM were uniform across pellets of each defecation. Coefficient of variations (CVs) for each defecation and median CV for each EIA were calculated.

### 2.5. Data Analyses

For each of the 3 (cortisol, 37e, 50c) EIAs, descriptive statistics were produced for FCM values, which included histograms for all samplings, boxplots for individual koalas, boxplots by age, sex and breeding season and line graphs showing the observed FCM mean (with 95% percentiles) per month. Normality of FCM values was assessed by visually examining the histograms and by using the Shapiro–Wilk test, followed by a log-transformation of the FCM values for further statistical analysis.

Mixed effect linear regression [52] was used to explore the association between log- transformed FCM values and predictor variables. Predictors included months of sampling, season (breeding season: September–January; non-breeding season: February–August), sex (male; female), age (categorised as: up to 2-years-old; between 2 years and 5 years; older than 5 years); and sampling time (morning; evening). The koala ID was included in the mixed effect linear regression model as a random effect to account for clustering of observations by koala. Model results were displayed as coefficients with 95% confidence intervals and the *p*-value. For categorical predictors with more than 2 levels, Wald-test *p*-values were calculated.

To explore seasonality, we decomposed the FCM values as times series. As our data represented a short time series with only one observation per seasonal period, we used Fourier terms to approximate the seasonal patterns and decompose the time series [53]. We then used the Augmented Dickey–Fuller test [54] to test the significance of the seasonal effect. A *p*-value less than 0.05 indicates that the time series is stationary, signifying that the seasonal effect is minimal.

The descriptive and regression ana lysis was performed in STATA 16.1 (StataCorp LLC, 4905 Lakeview Drive, College Station, TX 77845, USA), while the time series analysis was conducted in R version 4.0.2 (R Core Team, 2020). [55].

## 3. Results

### 3.1. FCM Variation within Defecations

The analyses of all pellets for 10 separate defecations showed a CV between 9.7% and 28.7% (median: 17.4%) with cortisol EIA, 8.4% and 17.7% (11.6%) with 37e EIA and 12.3% and 24.3% (17.6%) with 50c EIA.

### 3.2. Overview of FCM Values Measured by EIAs

The box whisker plots indicating the variation of FCM values for each of the 13 koalas, measured with the cortisol, 37e, and 50c EIAs are shown in Figure S1 (Supplementary Materials).

**Figure 1:**
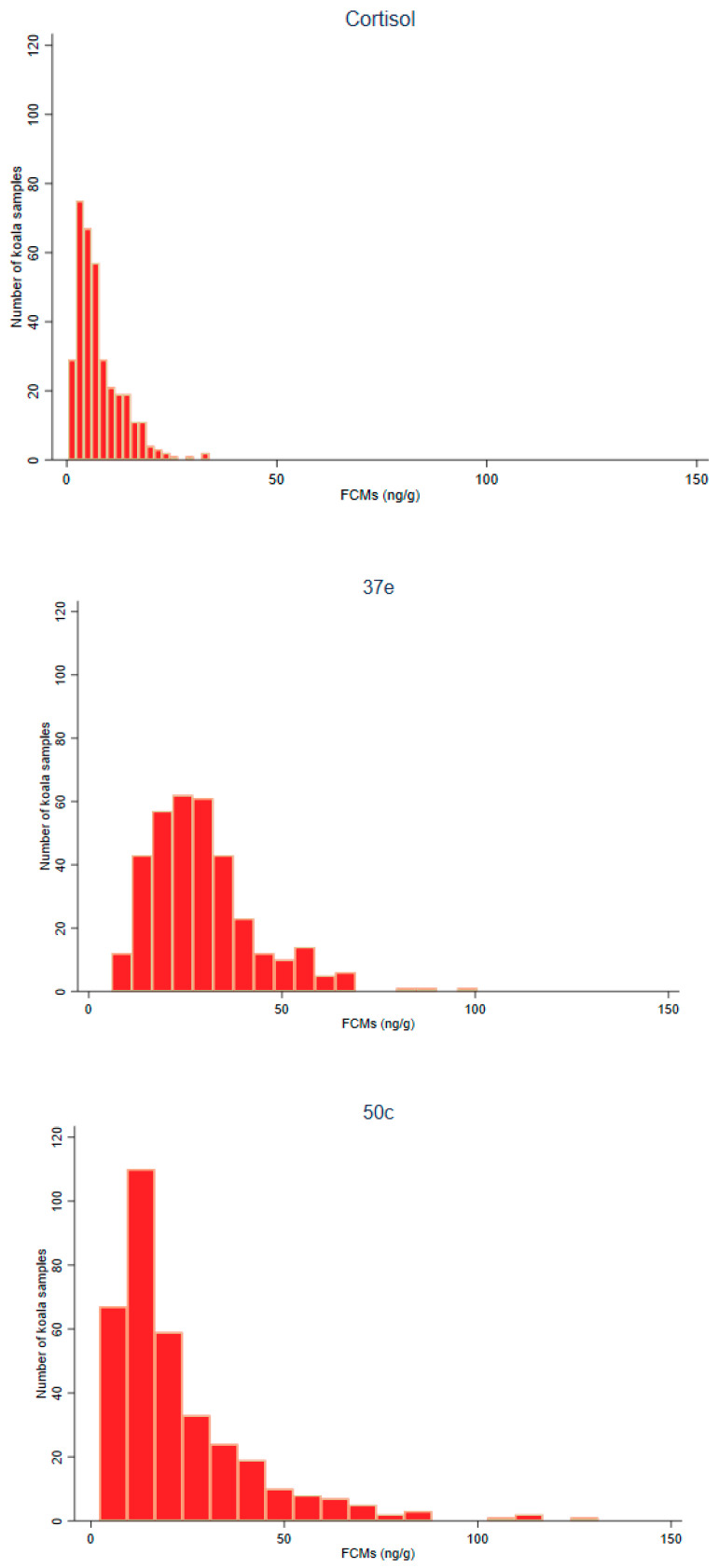
Histograms of FCM (faecal cortisol metabolite) values (ng/g) measured with the cortisol, 37e (5α-pregnane-3β,11β,21-triol-20-one) and 50c (tetrahydrocorticosterone) EIAs (enzyme immunoassays).

Histograms of the distribution of FCM values measured with the cortisol, 37e and 50c EIAs are shown in Figure 1. The mean, median, 25th, 75th percentile, interquartile range and minimum and maximum values for FCMs using a cortisol, 37e and 50c EIAs are shown in Table 2.

The measured FCM values were skewed for all three EIAs, with lowest median values for cortisol, followed by 50c and 37e. The widest interquartile range of values were detected with the 50c EIA, followed by 37e and cortisol EIAs.

### 3.3. FCMs by Sex, Age Categories and Time of Sampling

**Figure 2:**
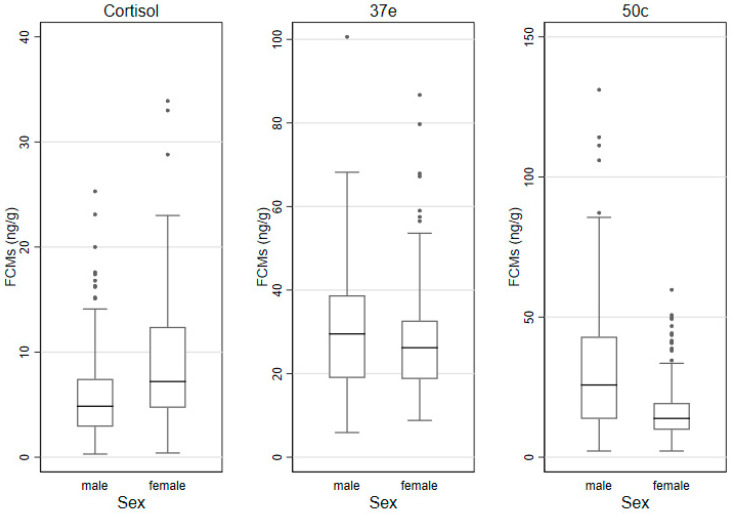
Box and whisker plots for FCM (faecal cortisol metabolite) levels (ng/g) measured with the cortisol, 37e (5α-pregnane-3β,11β,21-triol-20-one) and 50c (tetrahydrocorticosterone) EIAs (enzyme immunoassays), as grouped by sex (defecations: males *n*= 164; females *n*= 187).

**Figure 3:**
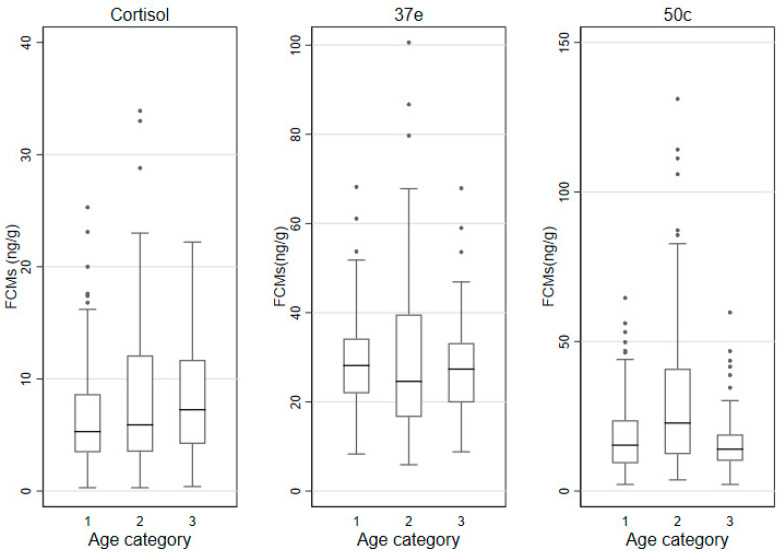
Box and whisker plots for FCM (faecal cortisol metabolite) levels (ng/g) measured with the cortisol, 37e (5α-pregnane-3β,11β,21-triol-20-one) and 50c (tetrahydrocorticosterone) EIAs (enzyme immunoassays), as grouped by age categories (1: ≤2 years-; 2: >2–5 years-; 3: >5 years; defecations *n*= 124, *n*= 159 and *n*= 68 respectively).

**Figure 4:**
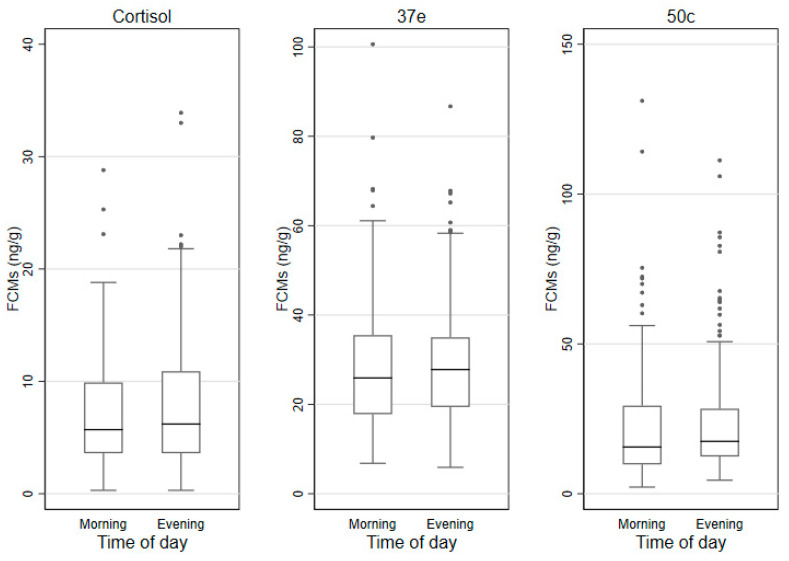
Box and whisker plots for FCM (faecal cortisol metabolite) levels (ng/g) measured with the cortisol, 37e (5α-pregnane-3β,11β,21-triol-20-one) and 50c (tetrahydrocorticosterone) EIAs (enzyme immunoassays) in morning and evening samples (defecations: *n* = 182 and *n* = 169 respectively).

The median (IQR) FCM values measured by the cortisol, 37e and 50c EIAs by sex, age category and time of sampling are shown in Table 3 and the box and whisker plots for these groups are shown in Figure 2, Figure 3 and Figure 4. In males, lowest/highest FCM values (ng/g) detected by the cortisol, 37e and 50c EIAs were 0.3/25.3; 5.9/100.6 and 2.2/131.1, respectively. In females, those lowest/highest FCM values were 0.4/33.9; 8.8/86.7 and 2.2/59.7, respectively.

### 3.4. Seasonality of FCM Values

**Figure 5:**
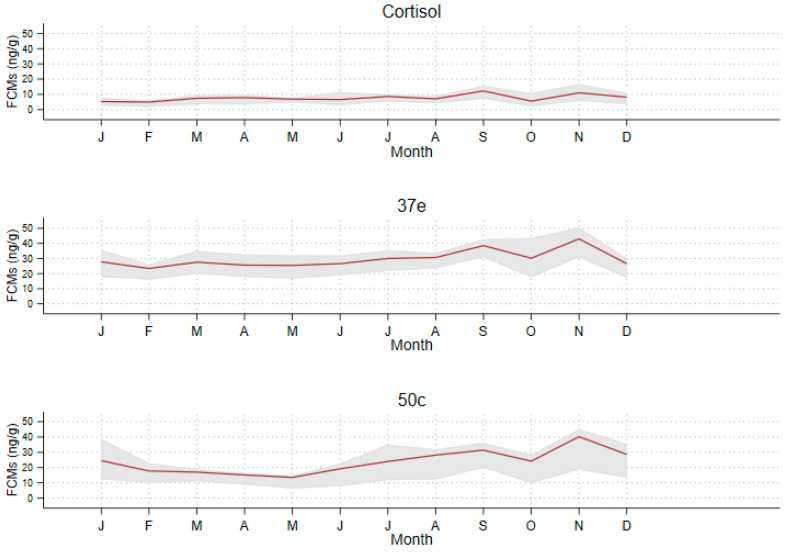
Monthly mean (red line) and 95% percentile (shaded area) FCM (faecal cortisol metabolite) values (ng/g) measured with the cortisol, 37e (5α-pregnane-3β,11β,21-triol-20-one) and 50c (tetrahydrocorticosterone) EIAs (enzyme immunoassays) (defecations: *n* = 351).

The mean (with 95% percentiles) FCM values measured per month are shown in Figure 5.

It is noticeable that there was a strong increase in FCM values detected by the three EIAs from September until January, coinciding with the breeding season of koalas (Figure 5). FCM values measured for each sampling interval are also shown in Figure S2a–c.

**Figure 6:**
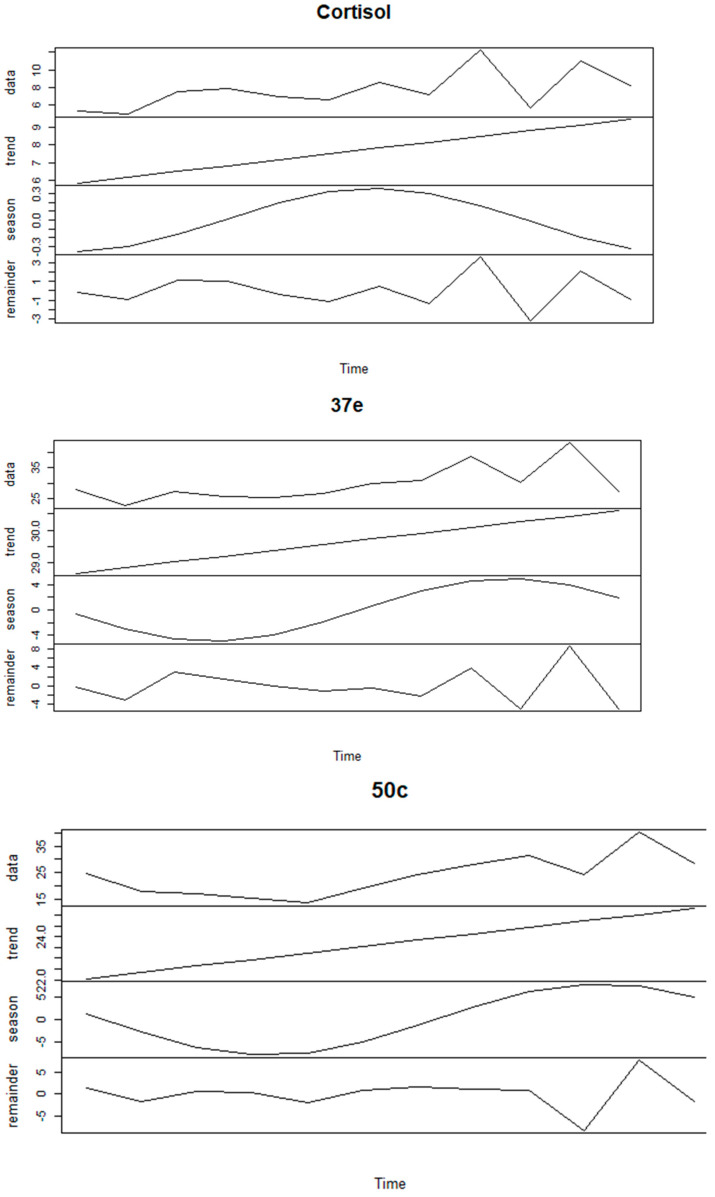
Time series for FCM (faecal cortisol metabolite) values measured with the cortisol, 37e (5α-pregnane-3β,11β,21-triol-20-one) and 50c (tetrahydrocorticosterone) EIAs (enzyme immunoassays) over a 12-month period.

We explored the seasonality of FCM values in more detail using time series analysis (Figure 6).

The decomposition of the time series dataset highlighted a strong seasonal trend for 37e (Augmented Dickey–Fuller test = −1.4224, *p*-value = 0.7924 for hypothesis of stationary time series) and 50c (Augmented Dickey–Fuller test = −2.1821, *p*-value = 0.503), but less so for cortisol (Augmented Dickey–Fuller test = −3.391, *p*-value = 0.079).

**Figure 7:**
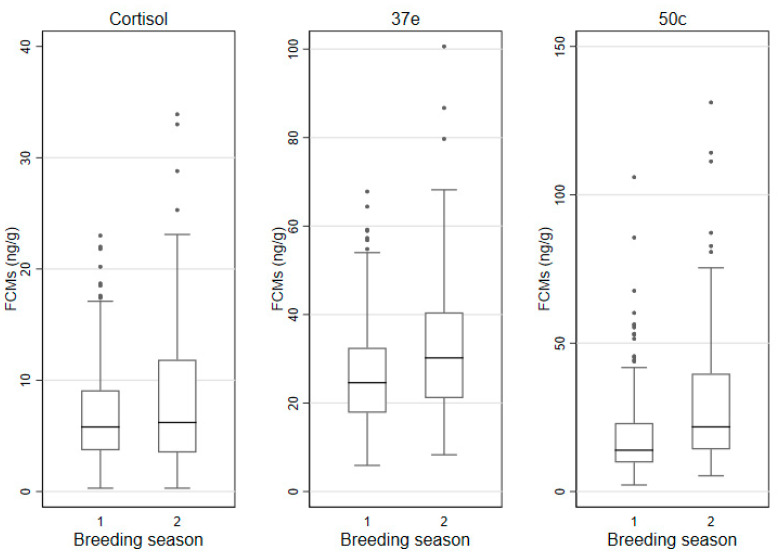
Box and whisker plots for FCM (faecal cortisol metabolite) values (ng/g) measured with the cortisol, 37e (5α-pregnane-3β,11β,21-triol-20-one) and 50c (tetrahydrocorticosterone) EIAs (enzyme immunoassays) (1: non-breeding season (February–August); 2: breeding season (September–January); defecations: *n* = 199 and *n* = 152 respectively).

The FCM values were then grouped by breeding and non-breeding season and displayed in box andwhisker plots (Figure 7), while the descriptive statistics are shown in Table 4.

### 3.5. Correlation of FCM Values between EIAs

**Figure 8:**
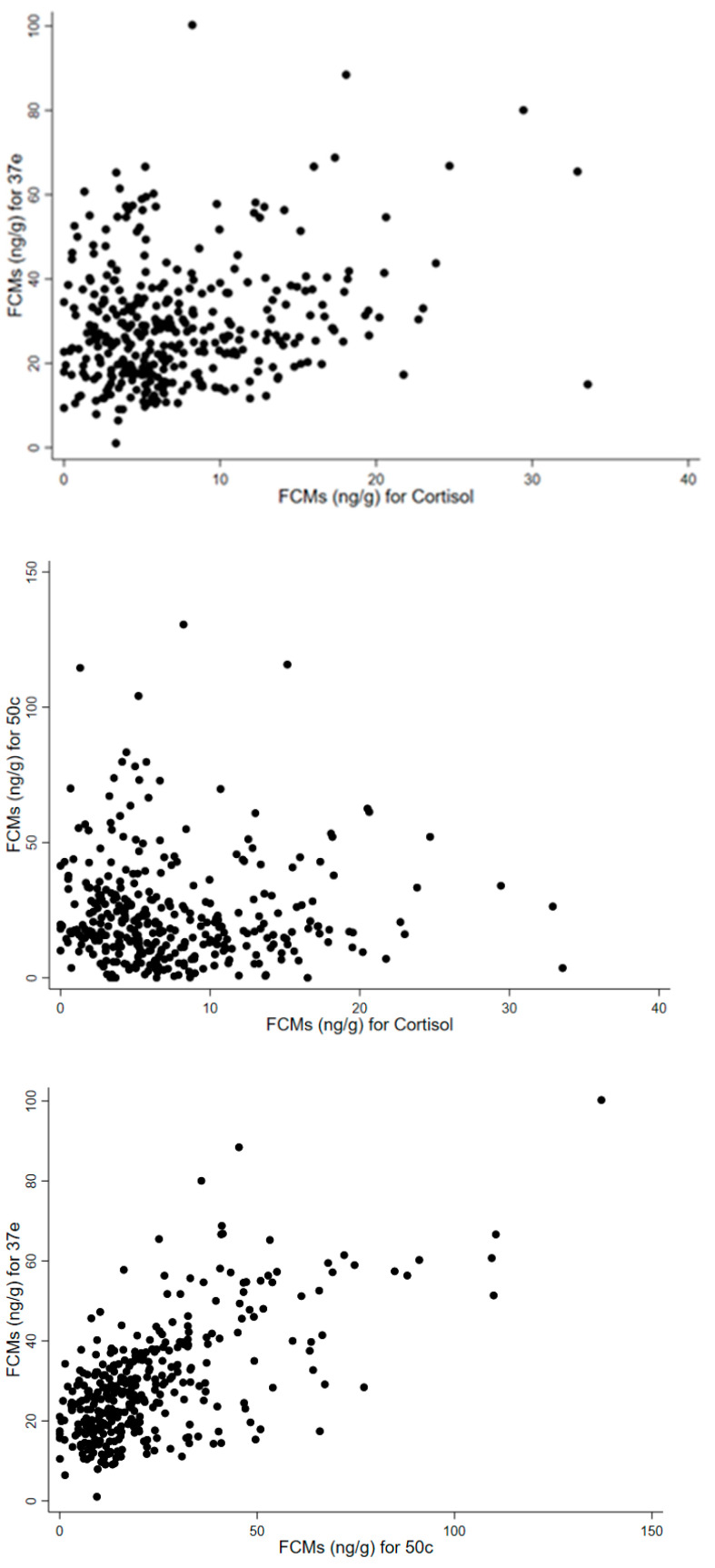
Scatterplots correlating FCM (faecal cortisol metabolite) values (ng/g) measured with cortisol, 37e (5α-pregnane-3β,11β,21-triol-20-one) and 50c (tetrahydrocorticosterone) EIAs (enzyme immunoassay) conducted over a period of 12 months (defecations: *n* = 351).

Scatterplots were used to display the bivariate correlations between groups of two EIAs (Figure 8).

The Pearson Correlation coefficient for 37e vs. cortisol was 0.2426 (*p* < 0.0001), for 50c vs. cortisol was −0.0039 (*p* = 0.9423) and for 37e vs. 50c was 0.6776 (*p* < 0.0001). Thus, a strong, significant correlation was only observed for the relationship between FCM concentrations measured with the 37e and 50c EIAs.

### 3.6. Factors Affecting FCM Values

The impact of predictors on the log-transformed FCM values was then evaluated in mixed effect linear regression models. In the univariate analysis, sex of the koalas affected log-transformed FCM values when measured with the 50c EIA: values of males were significantly higher (*p* = 0.022) than those of females. Sex differences were not detected with the cortisol or 37e EIAs. Age was not a factor affecting the values of log-transformed FCMs measured with any of the three EIAs. Log-transformed FCM values were significantly higher in the evening samples, when measured with the 50c EIA (*p* = 0.036). However, such differences were not detected with the other two EIAs. Log-transformed FCM values were significantly higher in the breeding season compared to the non-breeding season, when measured with the 50c EIA (*p* < 0.001). This association was also identified with the 37e EIA (*p* = 0.001), but not with the cortisol EIA. The univariate and multivariable analysis results are displayed in Table S1 (Supplementary material). There was no significant interaction between breeding season and sex of koalas in the multivariable model for the 50c EIA.

## 4. Discussion

Non-invasive methods for measuring FCMs are frequently utilized to investigate stress in animals [35]. However, not all EIAs can successfully measure FCMs in all species [40,46], hence biological performance was a priority here, to ensure the selection of the most appropriate EIA for koalas as the target species [26]. Whereas our previous study [47], identified FCMs and validated three EIAs for their measurement in koalas, the current one has tested the biological sensitivity of those three EIAs over a period of one year and determined the most suitable EIA for future studies on stress in koalas.

However, before monitoring stress, establishing baseline levels of cortisol metabolites is of paramount importance. As we assumed that FCM values would likely differ during the year, we conducted a longitudinal study on adrenocortical activity involving a confined koala population and have established FCM baseline values and the effect of time of day, the animals’ sex and age on these values and the seasonal FCM patterns during the non-breeding and breeding season of koalas. FCM baseline values were measured in stress-free koalas adapted to the presence of humans, kept in a constant environment with minimal external disturbance and consistent access to food and water. We only entered the enclosures to access the freshly defecated faecal pellets. We chose to carry out our study with a captive cohort to exclude all the factors that may be cause of stress in wild koalas and potentially increase the levels of FCMs.

The concept of baseline, when referring to glucocorticoid values, has been given different meaning depending on the type of research and the intent of the researchers [41,56,57], and in many cases refers to a point-in-time value [39,40,41] using a limited number of animals. Admittedly, the main intent of most of these studies was to physiologically validate EIAs before and after an ACTH challenge; however, some authors compare obtained FCM values to those of ‘stressed’ animals [41,58,59] without taking into consideration the physiological changes due to period of day, sex and season. Other year-long studies on marsupials were carried out on numbats and wombats also in captivity [38,60]. Yet, only the study on numbats [38] evaluated the variations of FCM values during breeding and non-breeding season, as well as differences between females and males. While we can draw some parallels with those studies, the term baseline is not comparable to ours, as human intervention during those research projects may have caused baseline values to be altered.

We found significantly higher FCM baseline values in males than in females when using the 50c EIA, but we did not detect differences between females and males with neither the cortisol nor the 37e EIAs. A previous study [41] on captive koalas over a 20-day period during the breeding season, found that there were sex differences in FCM values using a cortisol EIA. Yet, it appears that these values were obtained from various groups of males and females (handled, non-handled and with diverse reproductive status) and, therefore, it is not clear if these differences represented undisturbed stress-free koalas. Another study [58] on wild koalas reports no sex differences in FCM values with a similar type of EIA. The year-long study on numbats [38] using a cortisol EIA, reported higher FCM values for males during the breeding season, but not for females, despite a marginal increase. These animals were often handled and, therefore, the results may have been different if they had been left undisturbed. In our study, we did not find any significant relationship between FCM values during the breeding season and the sex of koalas.

Similarly, 50c was the only EIA detecting significant differences between morning and evening samples. We didn’t expect to detect any differences in circadian values due to the length of the caecum of koalas (2 m long or 23% of the intestinal length) where the broken down digested material is further metabolized by a large variety of bacteria [61]. In this section of the intestine, cortisol metabolites could be spread throughout the digested matter, potentially levelling out any peak values that may exist between morning and evening as Touma and Palme [24] found in the case of hind gut fermenters. They suggested that when collecting faecal samples of long gut passage fermenters, the time of collection needs to be considered as it may be otherwise difficult obtaining circadian values. However, there is the possibility that the different circadian values of FCM in this study may originate from the addition of cortisol (metabolites) secreted by the large intestine (colon) located after the caecum. A study [62] found that the epithelium of the colon is also capable of synthesizing GC in response to particular immune responses. While we cannot assert that the colon of koalas is also a source of cortisol metabolism, we suggest that this is something to be further investigated.

Our study did not find any differences in FCM values between the three age categories with any of the EIAs. There are no comparable studies on koalas, nevertheless, research on other marsupials (tammar wallabies) [63] showed no differences in blood cortisol values related to age in captured animals. Also, although physiologically different to marsupials, storks [64] show no age-related differences in baseline GC values. Yet, a study on dingoes [65] found that puppies had higher salivary cortisol values than adults. Therefore, we may speculate that age related differences in FCM values are likely to be species-specific.

Establishing average, minimum and maximum FCM baseline values was the main scope of this research as behavioural changes, during the breeding time as well as pregnancy itself [66], can lead to increased stress. In turn, higher levels of stress have been shown to have a negative effect on gonadotropin secretion, compromising reproduction, in many animal species [67], including humans. Baseline values need to be taken into account together with all the other parameters [24], because evaluating stress without previously establishing these baseline levels may potentially lead to errors in assessment.

Koalas’ breeding season may vary slightly across their northern–southern latitudinal range. Some authors have found that in South East Queensland the koala breeding season is between October and March and non-breeding is between April and September [68], while others found that females showed behavioural signs of oestrus mainly between September and April [69]. In particular, koalas at Wildlife HQ, where this study was conducted, mate during “September through to early/mid-February” (T. Maxwell personal communications). When we analysed monthly mean FCM values throughout the year we noticed that all three EIAs could detect an increase during the breading season (September–January). Yet, statistical analysis, in particular the time series analysis, revealed that only 37e and 50c EIAs could detect considerable differences between breeding and no-breeding season and we also identified a strong correlation of FCM values between these two EIAs. The 37e and 50c EIAs were originally designed to target glucocorticoid metabolites with a 5α-3β,11β-diol and 5β-3α,11β-diol molecular structure and were validated for rodents and birds, respectively [48,49]. Therefore, two metabolites, also found in the koala faeces, namely 3β-allotetrahydrocortisol and tetrahydrocortisol (THF), can also be measured by the 37e and 50c EIA, respectively [47]. Our study has demonstrated that these group-specific EIAs are better suited in measuring FCM values than the cortisol EIA. However, we have shown here that the 50c EIA has the best discerning ability and the capability of detecting a broader range of FCM values than the 37e EIA. Therefore, given the ability of the 50c EIA to detect THF (the main FCM in koalas) and its broader dynamic range of detection, we propose the use of the 50c EIA as the “koala-specific” assay.

We also observed some variation in FCM values for the three EIAs between individual pellets of one defecation. These CV values were in the range or somewhat higher than those for the pool sample extracts analysed in the EIAs. Therefore, whenever possible, we would advise to collect and homogenise at least two pellets from one defection to lower the influence of any potential variation. Faecal decay [70] and stability of FCMs [26,50] need to also be considered in future studies, especially when samples from unknown time of defecation are collected in the wild.

## 5. Conclusions

With this study, we found the tetrahydrocorticosterone (lab code: 50c) EIA best suited among three previously validated EIA for koalas. We have determined FCM baseline levels during the breeding and non-breeding season, differences between sexes and between morning and evening. Future studies on stress in koalas may now choose to compare their findings to our baseline values using this group-specific EIA, which can give consistent results.

## Figures and Tables

**Table 1:** Details of the EIAs (enzyme immunoassays) utilized in the current study.

EIA Code	Details	Description
Cortisol	Standard	4-pregnene-11β,17α,21-triol-3,20-dione(cortisol)
	Targeted structure	11β,17α,21-triol-20-one
	Antibody against	cortisol-3-CMO:BSA
	Label	cortisol-3-CMO-DADOO-biotin
	Reference	Palme and Möstl [51]
37e	Standard	5α-pregnane-3β,11β,21-triol-20-one (3β-allotetrahydrocorticosterone)
	Targeted structure	5α-3ß,11ß-diol
	Antibody against	5α-pregnane-3β,11β,21-triol-20-one-CMO-BSA
	Label	5α-pregnane-3β,11β,21-triol-20-one-CMO-biotinyl-LC
	Reference	Touma et al. [49]
50c	Standard	5β-pregnane-3α,11β,21-triol-20-one(tetrahydrocorticosterone)
	Targeted structure	5β-3α,11β-diol
	Antibody against	5β-pregnane-3α,11β,21-triol-20-one-CMO-BSA
	Label	5β-pregnane-3α,11β,21-triol-20-one-21-HS-biotinyl-LC
	Reference	Quillfeldt and Möstl [48]

**Table 2:** Summary statistics for FCM (faecal cortisol metabolite) levels (ng/g) measured by cortisol, 37e and 50c EIAs (enzyme immunoassays).

EIA	Mean	Median	p25	p75	IQR	Min	Max
Cortisol	7.6	5.9	3.6	10.6	7.0	0.3	33.9
37e	29.3	27.3	18.8	35.3	16.5	5.9	100.6
50c	23.3	16.5	11.1	29.4	18.3	2.2	131.1

p = percentiles, IQR = Inter Quartile Range; Min = minimum, Max = maximum.

**Table 3:** Median (IQR) for FCM (faecal cortisol metabolites) values measured by cortisol, 37e and 50c EIAs (enzyme immunoassays) by sex, age category and time of sampling.

Variable	Sub-Groups	FCM (ng/g)		
		Cortisol EIA	37e EIA	50c EIA
Sex	Male	5.9 (4.6)	31.0 (19.8)	31.1 (29.4)
	Female	9.0 (7.7)	27.9 (14.0)	16.4 (9.6)
Age	≤2 years	6.5 (5.2)	28.8 (12.3)	18.4 (14.4)
	>2–5 years	8.0 (8.6)	30.2 (23.0)	29.9 (28.6)
	>5 years	8.5 (7.5)	27.8 (13.6)	16.6 (8.9)
Time	Evening	8.1 (7.3)	29.5 (15.6)	24. 1 (16.0)
of day	Morning	7.1 (6.3)	29.1 (17.7)	22.5 (19.6)

37e (5α-pregnane-3β,11β,21-triol-20-one) and 50c (tetrahydrocorticosterone).

**Table 4:** Summary statistics for FCM (faecal cortisol metabolites) values measured with the cortisol, 37e and 50c EIAs (enzyme immunoassays) by breeding season.

EIA	Mean	Median	p25	p75	IQR
Non-breeding (February–August)					
Cortisol	7.0	5.8	3.7	9.1	5.4
37e	26.8	24.6	17.8	32.5	14.7
50c	18.7	13.9	9.8	23.1	13.3
Breeding (September–January)					
Cortisol	8.2	6.2	3.5	11.9	8.4
37e	32.7	30.2	21.1	40.5	19.4
50c	29.2	21.8	14.2	39.7	25.6

p = percentiles, IQR = Inter Quartile Range; Min = minimum, Max = maximum.

## Data Availability

Raw data are available from corresponding author upon reasonable request.

## Supplementary Materials

The following are available online at https://www.mdpi.com/article/10.3390/ani11061622/s1, Figure S1: Box and whisker plots of 13 koalas sampled for FCM levels (ng/g) using three EIAs (Cortisol, 37e, 50c). Figure S2: Fortnightly mean (red line) and 95% percentile (shaded area) FCM values (ng/g) measured with the cortisol, 37e and 50c EIA. Table S1: Results of mixed effect linear regression models (with koala ID as random effect) to explore the association between log-transformed FCM values for the cortisol, 37e and 50c EIA, respectively, and predictors (month, breeding season, time of day, sex, age category).
