# Supplementary material for: Seasonal Variations of Faecal Cortisol Metabolites in Koalas in South East Queensland
Source: Animals (Basel). 2021 May 31;11(6):1622. doi: 10.3390/ani11061622 (PMC8227722; doi:10.3390/ani11061622)
Supplement: Supplementary file 1 [file animals-11-01622-s001.zip › animals-1232336-SI.pdf]

Seasonal variations of faecal cortisol metabolites in koalas in South-East Queensland

Supplementary Information

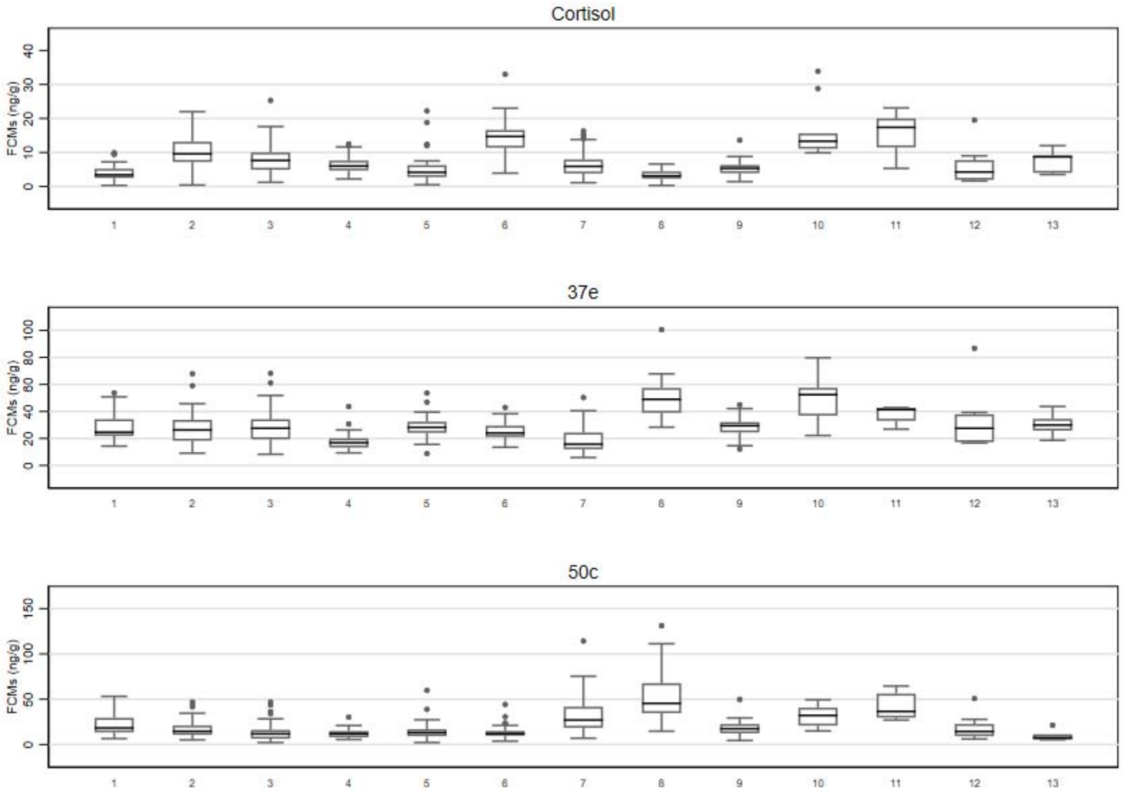

Figure S1: Box and whisker plots of 13 koalas sampled for FCM levels (ng/g) using three EIAs (Cortisol, 37e, 50c). Koalas 1, 2, 3 & 7 were sampled 46 times, 8 & 9 were sampled 40 times, 5 & 6 were sampled 38 times, 4 was sampled 34 times, 10 was sampled 12 times, 12 & 13 were sampled 8 times and 11 was sampled 6 times.

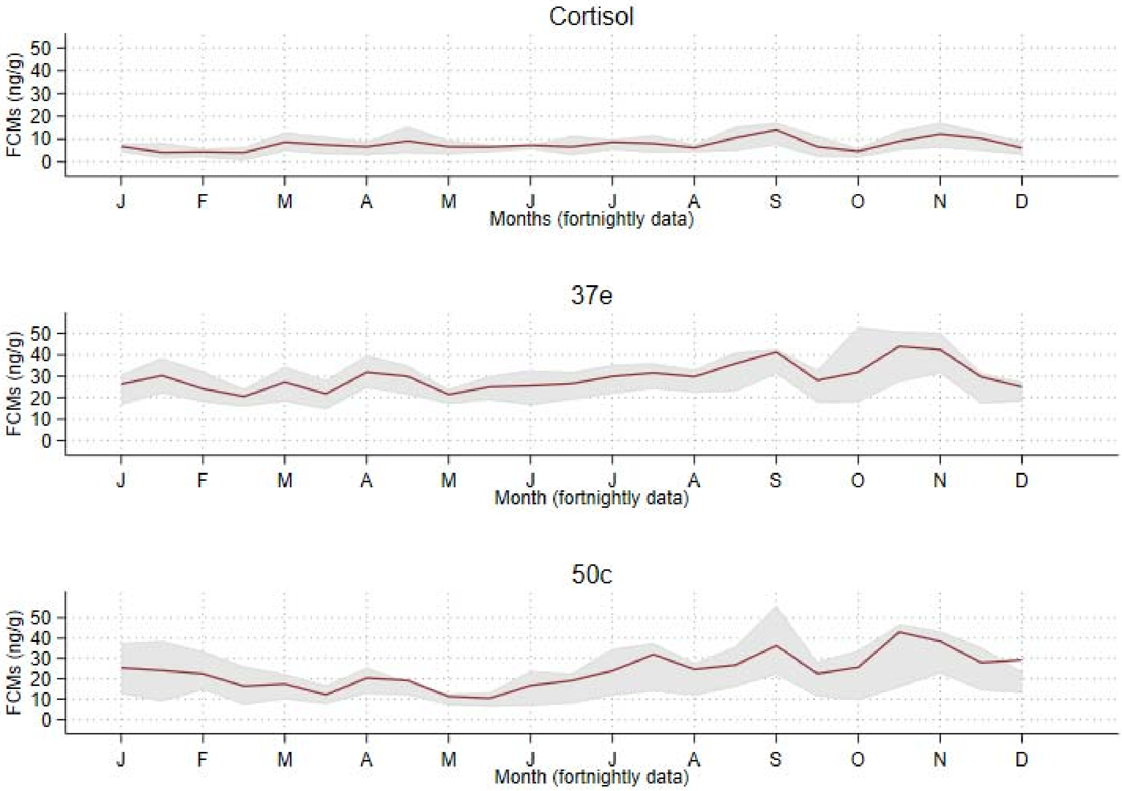

Figure S2: Fortnightly mean (red line) and 95% percentile (shaded area) FCM values (ng/g) measured with the cortisol, 37e and 50c EIA.

Table S1: Results of mixed effect linear regression models (with koala ID as random effect) to explore the association between log-transformed FCM values for the cortisol, 37e, and 50c EIA, respectively and predictors (month, breeding season, time of day, sex, age category. Please note due to collinearity with breeding season, month was not used in the multivariable model).

								**Univariate analysis**			**Multivariable analysis**	
		**Cortisol EIA (logFCM)**			**37e EIA (logFCM)**			**50c EIA (logFCM)**			**50c EIA (logFCM)**	
**Variable**	**Subgroups**	**Coefficient**	**p-value**	**Wald test p-value**	**Coefficient**	**p-value**	**Wald test p-value**	**Coefficient**	**p-value**	**Wald test p-value**	**Coefficient**	**p-value**
Month	Jan	0		<0.001	0		<0.001	0		<0.001		
	Feb	-0.23 (-0.52, 0.05)	0.108		-0.16 (-0.36, 0.03)	0.101		-0.27 (-0.60, 0.06)	0.114			
	Mar	0.37 (0.11, 064)	0.006		-0.01 (-0.19, 017)	0.921		-0.26 (-0.57, 0.05)	0.099			
	Apr	0.41 (0.14, 0.69)	0.003		-0.07 (-0.26, 0.12)	0.477		-0.43 (-0.75, -0.11)	0.009			
	May	0.37 (0.10, 0.64)	0.008		-0.07 (-0.26, 0.11)	0.439		-0.64 (-0.96, -0.32)	<0.001			
	Jun	0.19 (-0.14, 0.53)	0.254		-0.05 (-0.27, 0.18)	0.697		-0.33 (-0.72, 0.07)	0.103			
	Jul	0.55 (0.21, 0.90)	0.002		0.12 (-0.11, 0.35)	0.314		0.03 (-0.37, 0.43)	0.888			
	Aug	0.37 (0.09, 0.65)	0.010		0.12 (-0.11, 0.32)	0.203		0.12 (-0.21, 0.45)	0.479			
	Sep	0.67 (0.35, 0.99)	<0.001		0.37 (0.17, 057)	<0.001		0.32 (-0.02, 0.65)	0.062			
	Oct	-0.17 (-0.48, 0.14)	0.294		0.04 (-0.16, 0.24)	0.694		-0.03 (-0.36, 0.30)	0.857			
	Nov	0.68 (0.37, 0.98)	<0.001		0.45 (0.25, 0.66)	<0.001		0.48 (0.13, 0.84)	0.007			
	Dec	0.35 (0.08, 0.62)	0.012		-0.01 (-0.19, 0.18)	0.953		0.09 (-0.23, 0.41)	0.569			
Breeding season	Feb-Aug (non-breeding)	0.00			0			0			0	
	Sep-Jan (breeding)	-0.01 (-0.15, 0.13)	0.896		0.14 (0.22, 3.32)	0.001		0.45 (0.33, 0.57)	<0.001		0.47 (0.35, 0.59)	<0.001
Time of day	Morning	0			0			0			0	
	Evening	0.13 (-0.01, 0.26)	0.062		0.02 (-0.06, 0.10)	0.618		0.13 (0.01, 0.24)	0.036		0.17 (0.06, 0.28)	0.003
Sex	Male	0			0			0			0	
	Female	0.33 (-0.29, 0.94)	0.299		-0.05 (-0.42, 0.31)	0.772		-0.59 (-1.10, -0.09)	0.022		-0.62(-1.15, -0.10)	0.019
Age category	up to 2 years	0.00			0		0.939	0		0.559		
	>2 years to 5 years	0.03 (-0.69, 0.75)			-0.05 (-0.46, 0.36)	0.818		0.26 (-0.40, 0.92)	0.439			
	> 5 years	-0.01 (-0.99, 0.96)	0.995		-0.10 (-0.65, 0.46)	0.734		-0.17 (-1.07, 0.73)	0.708			
